# The Principles of Diagnosis

**Published:** 1839-02-16

**Authors:** 


### BIBLIOGRAPHICAL NOTICE.

*The Principles of Diagnosis.* By Marshall Hall, M. D., F. R. S. *London and Edinburgh Edition, etc. Second American Edition, with Notes.* By John A. Swett, M. D. pp. 458. New York: D. Appleton & Co. 1839.

The works of Dr. Hall are well known to many of our readers,—that on Diagnosis is not amongst the least familiar of them. The present edition is published under the inspection of Dr. Swett, one of the best educated physicians of New York, and one well able to appreciate the importance of rendering the study of accurate diagnosis popular amongst his countrymen. The editor has added a number of valuable notes, and expresses his obligations to Dr. Bulkley, of New York, for the additions relative to diseases of the skin.

Hall on Diagnosis has been so long before the public, that it would be misplaced for us to speak of the merits of the work. The just evidence of its value, is the approbation which it has received from the public, sustaining the work through several successive editions. But we are glad to express the satisfaction we feel at the evidently increasing attention which the subject is receiving. It is but a few years since, that it was regarded as at least unnecessary, if not ludicrous, to attempt a minute diagnosis of the numerous varieties of disease, and of the organic lesions which occur during their course. This is no longer the case;—most physicians are convinced that accurate diagnosis is always useful, and often essential to the correct management of diseases, and that without the most diligent attention to the distinctive characters of disease, we can never arrive at the knowledge of pathology which will point out to us the exact limits of our art.

It should not, however, be forgotten, that diagnosis is at present necessarily imperfect,—and a work like that under consideration can do nothing more than assist us in acquiring the signs of disease, as far as they are at present known. It is a useful guide to those who possess a general acquaintance with disease, and presents to them the symptoms grouped together in such a way as to be most convenient for application. It must not be regarded as a work on disease in general, but simply as a means of retaining our knowledge, and of keeping it in readiness for use.

